# Trichosanthis Semen Suppresses Lipopolysaccharide-Induced Neuroinflammation by Regulating the NF-κB Signaling Pathway and HO-1 Expression in Microglia

**DOI:** 10.3390/toxins13120898

**Published:** 2021-12-14

**Authors:** Seungmin Lee, In Gyoung Ju, Yujin Choi, Sangsu Park, Myung Sook Oh

**Affiliations:** 1 Department of Biomedical and Pharmaceutical Sciences, Graduate School, Kyung Hee University, Seoul 02447, Korea; smlee0817@khu.ac.kr (S.L.); yj001217@khu.ac.kr (Y.C.); 2 Department of Life and Nanopharmaceutical Sciences, Graduate School, Kyung Hee University, Seoul 02447, Korea; igju801@khu.ac.kr; 3 Department of Fundamental Pharmaceutical Sciences, Graduate School, Kyung Hee University, Seoul 02447, Korea; x-zara@nate.com; 4 Department of Oriental Pharmaceutical Science, College of Pharmacy, Kyung Hee East-West Pharmaceutical Research Institute, Kyung Hee University, Seoul 02447, Korea

**Keywords:** trichosanthis semen, nuclear factor kappa B, interleukin-10, heme oxygenase 1, neuroinflammation

## Abstract

Neuroinflammation, which is mediated by microglia that release various inflammatory cytokines, is a typical feature of neurodegenerative diseases (NDDs), such as Alzheimer’s disease and Parkinson’s disease. Hence, alleviating neuroinflammation by downregulating pro-inflammatory action, and upregulating anti-inflammatory action of microglia is an efficient therapeutic target for NDDs. In this study, we evaluated whether trichosanthis semen (TS), a dried ripe seed of *Trichosanthes kirilowii* Maximowicz, reduces lipopolysaccharide (LPS)-induced neuroinflammation by regulating microglial responses in vitro and in vivo. Our results presented that TS reduced the release of pro-inflammatory mediators, such as nitric oxide (NO), inducible NO synthase, tumor necrosis factor-α, interleukin-1β, and interleukin-6 via inhibition of the nuclear factor kappa B (NF-κB) signaling pathway in LPS-treated BV2 microglial cells. Moreover, TS induced anti-inflammatory mediators, such as interleukin-10, found in inflammatory zone 1, and chitinase 3-like 3 by the upregulation of heme oxygenase 1 (HO-1). We further confirmed that TS administration suppressed microglial activation, but enhanced HO-1 expression in LPS-injected mice. These results suggest that TS has anti-neuroinflammatory effects via inhibition of NF-κB signaling through the activation of HO-1, and that TS may be a therapeutical candidate for NDDs treatment.

## 1. Introduction

Neuroinflammation, an inflammatory response within the central nervous system (CNS), induces neurodegenerative diseases (NDDs), such as Alzheimer’s disease and Parkinson’s disease [1,2]. Microglia, which play a crucial role like macrophages in the CNS, mediate both innate and acquired immune systems [3]. Excessive microglia activation induced by inflammatory stimuli, such as lipopolysaccharides (LPS), releases various inflammatory factors, including nitric oxide (NO), tumor necrosis factor-α (TNF-α), interleukin-1β (IL-1β), and IL-6 [4,5]. In addition, overactivated microglia aggravate neuroinflammation, and induce neuronal death in the CNS [6]. Hence, the inhibition of microglial activation would be an efficient therapeutic target in preventing NDDs.

The nuclear factor kappa B (NF-κB) pathway modulates microglial activation, and increases the levels of inducible NO synthase (iNOS) and cytokines, such as TNF-α, IL-1β, and IL-6, which induce neurotoxicity [7,8,9]. On the other hand, heme oxygenase-1 (HO-1) inhibits inflammation by regulating the NF-κB pathway, and inducing anti-inflammatory cytokines, such as IL-10 and transforming growth factor-β (TGF-β) [7,10,11,12]. These cytokines increase arginase 1, found in inflammatory zone 1 (FIZZ1), and chitinase 3-like 3 (Ym-1), which suppress inflammation, and repair tissue damage [13]. Because NF-κB and HO-1 are crucial mediators in regulating neuroinflammation, modulating NF-κB and HO-1 can be efficient in preventing neuroinflammation.

Trichosanthis semen (TS) is a dried ripe seed of *Trichosanthes kirilowii* Maximowicz (Cucurbitaceae), which is a traditional medicine that has been used as a cough medicine and expectorant [14]. In addition, TS contains a large amount of lipids, such as triglycerides, glycolipids, phospholipids, and chlorophyll [14]. Recently, it was reported that TS has anti-inflammatory and anti-tumor effects [15,16]. The various active compounds of TS include trichokitin-S1, α-kirilowin, β-kirilowin, and lectin; particularly, triterpenes, such as 3-epikarounidiol, 7-oxoisomultiflorenol, and 3-epibryonolol, were documented for their effects on inflammation [17,18,19,20,21]. However, the effect of TS on neuroinflammation has not yet been explored.

In this study, we aimed to evaluate whether TS extract (TSE) can reduce LPS-induced neuroinflammatory responses in BV2 microglial cells or in mouse brains. First, the expression of pro-inflammatory factors was assessed including NO, iNOS, TNF-α, IL-1β, and IL-6, and the phosphorylation of NF-κB in BV2 microglial cells. Then, it was evaluated whether TSE regulates anti-inflammatory factors, such as IL-10, FIZZ-1, Ym-1, and HO-1. Lastly, anti-inflammatory effects of TSE were examined by analyzing microglial activation in LPS-injected mouse brains.

## 2. Results

### 2.1. Effects of TSE on NO Production and iNOS Expression in LPS-Treated BV2 Microglial Cells

**Figure 1:**
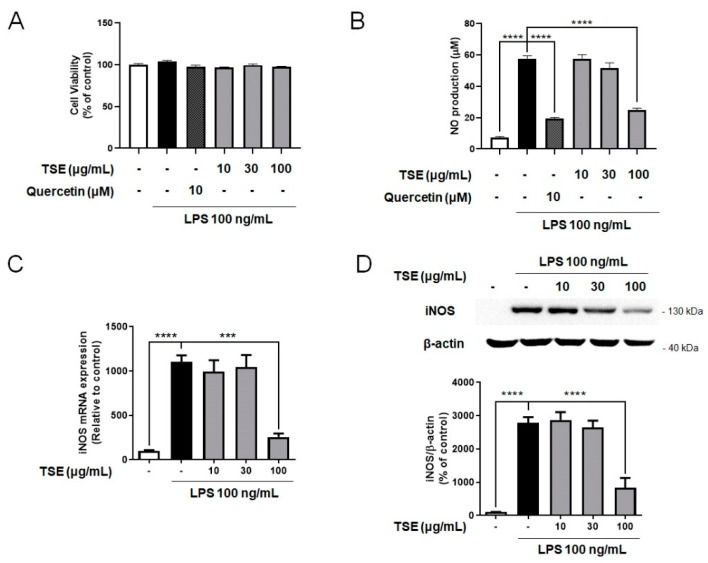
Effects of TSE on NO production, cell viability, and the expression levels of iNOS in BV2 microglial cells. The cells were pre-treated with TSE (10, 30, or 100 μg/mL) or quercetin (10 μM) for 1 h before LPS treatment for 23 h. Cell viability was assessed by MTT assay (**A**). The cell culture supernatant containing NO was evaluated using Griess reagent (*n* = 3 per group) (**B**). The mRNA levels of iNOS were quantified using qRT-PCR (*n* = 3 per group) (**C**). GAPDH was used as an internal control. The protein levels of iNOS were measured using western blotting in BV2 cell lysates. The representative band of iNOS is shown (**D**). The quantification of iNOS level was normalized to β-actin (*n* = 4 per group). Data were analyzed by one-way ANOVA, followed by Dunnett’s post hoc test. *** *p* < 0.001 and **** *p* < 0.0001.

NO released from microglia is an inflammatory mediator, and its overproduction induces neurotoxicity, as well as inflammation [22,23]. To evaluate whether TSE reduces NO production, we measured NO levels after treating BV2 microglial cells with TSE at the various concentrations with or without LPS. The current study used quercetin as a positive control due to its well-known anti-inflammatory properties [24]. It was found that the group pre-treated with TSE at 100 μg/mL showed significantly less NO levels, which were elevated in the LPS-treated group (Figure 1A,B). Moreover, under the LPS pre-treatment condition, TSE at 100 μg/mL decreased the level of NO production (Supplementary Figure S1).

To determine how TSE suppressed NO production, mRNA expression and protein levels of iNOS were evaluated, as iNOS is a major factor in the production of NO [25]. It was found that LPS treatment increased the mRNA expression and protein levels of iNOS compared to the control group, whereas TSE at 100 μg/mL remarkably suppressed iNOS expression in LPS-treated group (Figure 1C,D).

### 2.2. Effects of TSE on the Expressions of Pro-Inflammatory Cytokines in LPS-Treated BV2 Microglial Cells

**Figure 2:**
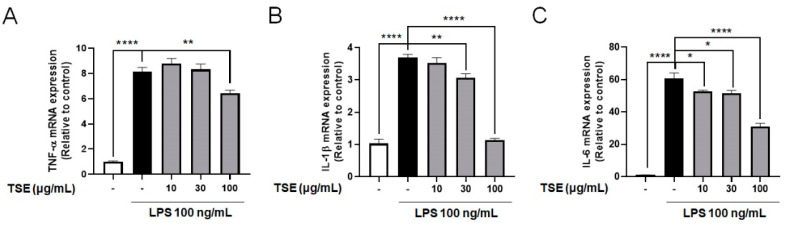
Effects of TSE on the mRNA levels of pro-inflammatory cytokines in BV2 microglial cells. The cells were pre-treated with TSE (10, 30, or 100 μg/mL) for 1 h before LPS treatment, and were treated with LPS for 23 h. The mRNA levels of TNF-α (**A**), IL-1β (**B**), and IL-6 (**C**) were quantified using qRT-PCR (*n* = 5 per group). GAPDH was used as an internal control. Data were analyzed by one-way ANOVA, followed by Dunnett’s post hoc test. * *p* < 0.05, ** *p* < 0.01, and **** *p* < 0.0001.

Pro-inflammatory cytokines from activated microglia could damage CNS tissue or neurons [5,26]. To assess whether TSE alleviates the increase in pro-inflammatory cytokines, mRNA expression of inflammatory cytokines was measured in LPS-treated BV2 microglial cells. It was found that the relative expression levels of pro-inflammatory cytokines were increased following LPS treatment. However, TSE treatment remarkably suppressed TNF-α, IL-6, and IL-1β at the mRNA level in a concentration-dependent manner (Figure 2).

### 2.3. Effects of TSE on NF-κB Signaling Pathway in LPS-Treated BV2 Microglial Cells

**Figure 3:**
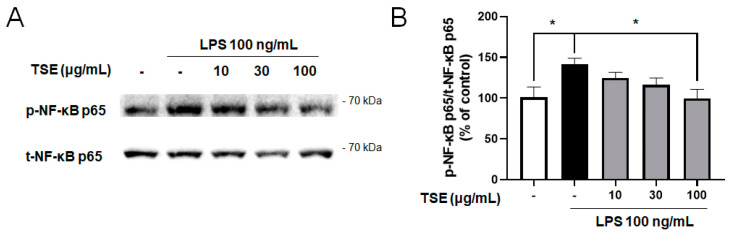
Effect of TSE on LPS-induced phosphorylation of NF-κB in BV2 microglial cells. The cells were pre-treated with TSE (10, 30, or 100 μg/mL) for 1 h before LPS treatment, and were treated with LPS for 30 min. The phosphorylated protein levels of NF-κB p65 were measured using western blotting in BV2 cell lysates. The representative band (**A**) and the quantification of phosphorylated-form/total form ratio of NF-κB p65 are shown (*n* = 5 per group) (**B**). Data were analyzed by one-way ANOVA, followed by Dunnett’s post hoc test. * *p* < 0.05.

NF-κB is a transcription factor that upregulates inflammatory responses. When NF-κB is phosphorylated, NF-κB translocates from the cytosol to the nucleus, and upregulates the expression of pro-inflammatory factors [27]. To evaluate whether TSE reduces the NF-κB activation, the levels of the phosphorylated and total form of NF-κB p65 were measured. The result showed that LPS treatment elevated the ratio of phosphorylated NF-κB p65. However, it was significantly suppressed by the TSE at 100 μg/mL (Figure 3B).

### 2.4. Effects of TSE on Expressions of IL-10, FIZZ1 and Ym-1 in LPS-Treated BV2 Microglial Cells

**Figure 4:**
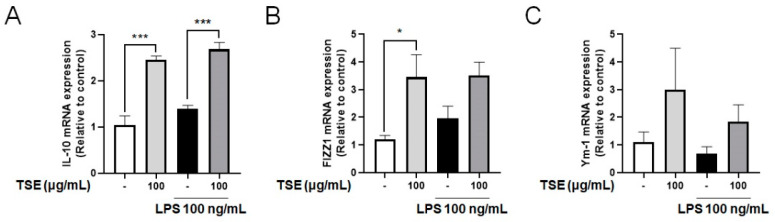
Effects of TSE on the mRNA levels of IL-10, FIZZ1, and Ym-1 in BV2 microglial cells. The cells were pre-treated with TSE at 100 μg/mL for 1 h before LPS treatment, and were treated with LPS for 23 h. The mRNA levels of IL-10 (**A**), FIZZ1 (**B**), and Ym-1 (**C**) were quantified using qRT-PCR (*n* = 3 per group). GAPDH was used as an internal control. Data were analyzed by one-way ANOVA, followed by Dunnett’s post hoc test. * *p* < 0.05 and *** *p* < 0.001.

The anti-inflammatory cytokine, IL-10, suppresses the pro-inflammatory responses, and upregulates FIZZ1 and Ym-1 levels, which then inhibits inflammation [13,28]. To assess whether TSE increases the expression of IL-10, mRNA expression of IL-10 was measured in LPS-treated BV2 microglial cells. Although no significant difference was observed between the control group and the LPS-treated group, TSE treatment significantly elevated the mRNA expression of IL-10 (Figure 4A). In addition, it was observed that the expression pattern of FIZZ1 and Ym-1 mRNA were similar to that of IL-10 when the cells were treated with TSE (Figure 4B,C).

### 2.5. Effects of TSE on Expressions of HO-1 in LPS-Treated BV2 Microglial Cells

**Figure 5:**
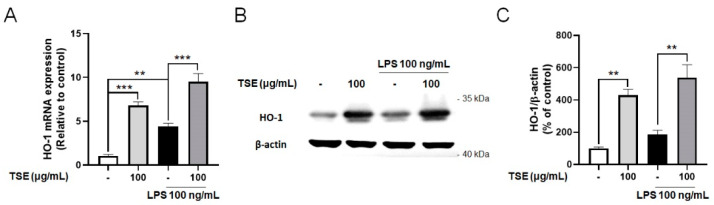
Effects of TSE on the expression of HO-1 in BV2 microglial cells. The cells were pre-treated with TSE at 100 μg/mL for 1 h before LPS treatment, and were treated with LPS for 23 h. The mRNA levels of HO-1 (**A**) were quantified using qRT-PCR (*n* = 3 per group). GAPDH was used as an internal control. The protein levels of HO-1 were measured using western blotting in BV2 cell lysates. The representative band of HO-1 is shown (**B**). Quantification of HO-1 level was normalized to β-actin (*n* = 3 per group) (**C**). Data were analyzed by one-way ANOVA, followed by Dunnett’s post hoc test. ** *p* < 0.01, *** *p* < 0.001.

HO-1 is known to suppress neuroinflammation, and protect neuronal cells by regulating pro-inflammatory factors [12,29]. To evaluate whether TSE upregulates the expression of HO-1, we measured the expressions of HO-1 in LPS-treated BV2 microglial cells. Contrary to the control group, the mRNA levels of HO-1 were elevated by TSE treatment at 100 μg/mL regardless of LPS treatment (Figure 5A). Furthermore, the expression pattern of HO-1 at the protein level was similar to that at the mRNA levels (Figure 5B,C).

### 2.6. Effects of TSE on Microgliosis and HO-1 Expression in LPS-Injected Mice

**Figure 6:**
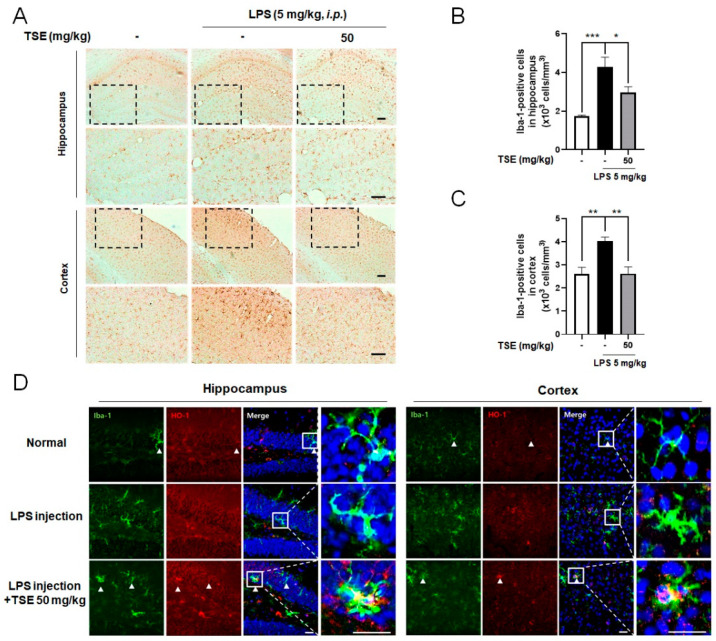
Effects of TSE on LPS-induced microglial activation in hippocampus and cortex. Mice were treated with TSE (50 mg/kg, p.o.) for 5 days, and injected with LPS (5 mg/kg, i.p.) on the fifth day. Representative images stained with DAB, and quantifications for the Iba-1^+^ cells in the hippocampus and cortex are shown (*n* = 5 per group) (**A**–**C**). The images of double immunofluorescence staining with anti-Iba-1 and anti-HO-1 in the hippocampus and cortex are shown (**D**). Scale bar = 100 μm. Data were analyzed by one-way ANOVA, followed by Dunnett’s post hoc test. * *p* < 0.05, ** *p* < 0.01, and *** *p* < 0.001.

To determine whether TSE suppresses neuroinflammation in vivo, we administered TSE to an LPS-injected mice model [30]. The number of ionized calcium-binding adapter molecule-1 (Iba-1)-positive cells was counted to test microglial activation in the mouse brain. Iba-1^+^ cells were increased in the LPS-injected group compared to the normal group in the hippocampus and cortex. However, TSE at 50 mg/kg significantly decreased the number of Iba-1^+^ cells (Figure 6A–C). Furthermore, to examine the expression of HO-1 in microglia, double immunofluorescence with anti-Iba-1 and anti-HO-1 was utilized. The result showed that the Iba-1-positive areas were considerably co-localized with the HO-1 positive area in the brain in TSE treatment compared to that of the LPS group (Figure 6D).

## 3. Discussion

In this study, we demonstrated the inhibitory effect of TS on LPS-induced neuroinflammation. In the LPS-treated BV2 microglial cells, TS reduced the release of pro-inflammatory factors, including NO, iNOS, TNF-α, IL-1β, and IL-6, by downregulating the NF-κB signaling pathway. Moreover, TS increased anti-inflammatory mediators, such as IL-10, FIZZ1, and Ym-1, by upregulating the HO-1 expression. In LPS-injected mice, TS alleviated the activation of microglia in the mouse brain. These results suggest that TS can inhibit neuroinflammation by regulating the pro-/anti-inflammatory responses of microglia.

In general, activated microglia stimulated by LPS are known to be neurotoxic, and are classified as M1 type microglia [31]. In the CNS, M1 type microglia release neurotoxic factors, such as NO and pro-inflammatory cytokines, which accelerate neuroinflammation. Conversely, M2 type microglia stimulated by interleukin-4 or interleukin-13 repair brain tissue damage, enhance neurotrophic factors, and reduce neuroinflammation. In addition, IL-10 increases the polarization of M2 type microglia, and the expression of M2 specific markers [13,32]. In this study, TS suppressed the expression of M1 cytokines, such as TNF-α, IL-1β, and IL-6, and increased the expression of M2 cytokines, such as IL-10, indicating that TS can decrease neurotoxic factors, but increase anti-inflammatory factors, and consequently suppress inflammatory responses in the brain.

HO-1 is considered as a crucial factor in the microglial anti-inflammatory processes. HO-1 produces biliverdin, carbon monoxide, and ferrous iron (Fe^2+^) by heme degradation [33,34]. Among the products of HO-1, carbon monoxide inhibits the NF-κB signaling pathway, reduces the expression of pro-inflammatory mediator, and modulates microglial activation [35,36]. In addition, all of the products of HO-1 can restrain the expression of iNOS and cyclooxygenase-2 [37,38,39,40]. At the same time, HO-1 promotes the expression of anti-inflammatory cytokines, such as IL-10, which has neuroprotective effects [41,42,43]. In the current study, TS elevated the HO-1 levels, resulting in anti-neuroinflammatory effects.

Akihisa et al. found that TS contains triterpenes, such as 3-epikarounidiol, 7-oxoisomultiflorenol, and 3-epibryonolol, which have been reported to reduce inflammations induced by 12-O-tetradecanoylphorbol-13-acetate in mice [21]. In addition, it has been reported that trichosanic acid, a common type of triglyceride, accounts for the highest content in TS, and affects chronic inflammation in 3T3-L1 cells [14,44]. From these previous reports, we hypothesize that the compounds previously mentioned can contribute to the downregulation of neuroinflammation. However, there have been no reports on which compounds can regulate neuroinflammation in microglial cells by modulating the expression of HO-1 and IL-10. Therefore, further studies elucidating the major compounds that play key roles in inducing anti-inflammatory responses in TS are necessary.

Neuroinflammation is a typical pathological feature of NDDs [45]. In Alzheimer’s disease or Parkinson’s disease, M1 type microglia are overactivated by pathogenic protein aggregates, such as beta-amyloid or alpha-synuclein, and produce inflammatory cytokines that induce neuronal cell death [32,46,47]. On the other hand, M2 type microglia degrade these misfolded proteins, and repair neuronal cell damage [48,49,50]. Thus, regulating the M1/M2 microglial phenotype to suppress neuroinflammation can be an effective approach to treat NDDs. Because TS showed beneficial effects on neuroinflammation, TS may be a promising therapeutic candidate for NDDs.

In conclusion, TS has anti-neuroinflammatory effects in LPS-induced neuroinflammation not only by downregulating the NF-κB signaling pathway, but by upregulating HO-1 expression. Therefore, these findings imply that TS can be a potential anti-neuroinflammatory candidate that prevent NDDs aggravated by neuroinflammation.

## 4. Materials and Methods

### 4.1. Materials

Dulbecco’s Modified Eagle Medium (DMEM), penicillin-streptomycin (P/S), and fetal bovine serum (FBS) were purchased from Hyclone Laboratories, Inc. (Logan, UT, USA). Rabbit anti-NF-κB p65 and mouse anti-β-actin were purchased from Santa Cruz Biotechnology (Temecula, CA, USA). Rabbit anti-p-NF-κB p65 was purchased from Cell Signaling Technology (Danvers, MA, USA). Goat anti-Iba-1 was purchased from Millipore Bioscience Research (Bedford, MA, USA). Rabbit anti-iNOS was purchased from Novus Biologicals (Littleton, CO, USA). Skim milk was purchased from BD Transduction Laboratories (Franklin Lakes, NJ, USA). Polyvinylidene difluoride (PVDF) was purchased from Millipore (Burlington, MA, USA). Chicken anti-goat Alexa 488 and goat anti-rabbit Alexa Fluor 594 were purchased from Invitrogen (Carlsbad, CA, USA). Normal chicken serum, normal goat serum, avidin-biotin complex (ABC) mixture, and anti-fade fluorescent mounting medium containing 4′,6-diamidino-2-phenylindole (DAPI) were purchased from Vector Laboratories (Burlingame, CA, USA). Rabbit anti-HO-1 and anti-rabbit horseradish peroxidase (HRP) secondary antibodies were purchased from Enzo Life Sciences, Inc. (Farmingdale, NY, USA). Tetramethylethylenediamine, protein assay reagent, acrylamide, enhanced chemiluminescence (ECL) reagent, protein standards dual color, and Tween 20 were purchased from Bio-Rad Laboratories (Hercules, CA, USA). Radio-immunoprecipitation assay (RIPA) buffer and protease/phosphatase inhibitor cocktail were purchased from Thermo Fisher Scientific (Waltham, MA, USA). Hybrid-R™ was purchased from GeneAll (Seoul, Korea). TOPscript™ RT DryMIX and TOPreal™ qPCR 2X PreMIX were purchased from Enzynomics (Daejeon, Korea). 3-(4,5-dimethyl-2-thiazolyl)-2,5-diphenyl-2H-tetrazolium bromide (MTT) and all the other reagents were purchased from Sigma-Aldrich (St. Louis, MO, USA), unless otherwise noted.

### 4.2. Preparation of TS Extract

TS was obtained from Naemomedah (Kwangmyoungdang Medicinal Herbs, Ulsan, Korea). The voucher specimen (BON20111701) of TS was deposited in the herbarium of the College of Pharmacy, Kyung Hee University (Seoul, Korea). The dried seeds were then boiled with 70% ethanol for 2 h. Next, the extract was filtered, evaporated on a rotary vacuum evaporator, and freeze-dried. The powder (yield: 0.69%) was maintained at 4 °C. The extract was resuspended in a proper vehicle prior to each experiment.

### 4.3. UPLC/ESI/MS Identification

Chromatographic analysis was performed with a Waters Acquity^TM^ ultra-performance liquid chromatography (UPLC) system (Waters Co., Milford, PA, USA) directly coupled to a Quattro micro^TM^ mass spectrometer (MS) system (Micromass Inc., Manchester, UK), which was equipped with an Acquity BEH C_18_ column (50 mm × 2.1 mm, 1.7 µm). 3,29-dibenzoyl karounitriol (ChemFaces, Wuhan, China) is a marker compound mainly contained in the TS [51]. The mobile phases for 3,29-dibenzoyl karounitriol were separated under isocratic elution with acetonitrile (Burdick&Jackson, Muskegon, MI, USA), with 0.1% formic acid (Wako, Osaka, Japan) and 0.4 mL/min. The column temperature was conditioned at 40 °C, and 2.0 µL was injected into the UPLC system, with an entire run time of 10 min. Chromatograms analyzed by UPLC/ESI/MS are shown in Supplementary Figure S2.

### 4.4. Cell Culture and Treatment

BV2 cells were maintained in DMEM supplemented with 10% FBS and 1% P/S, and incubated at 37 °C in a humidified atmosphere containing 5% CO_2_. All experiments were performed 24 h after seeding in 96-well plates (2.0 × 10^4^ cells/well) or in 6-well plates (1.0 × 10^6^ cells/well). After approximately 70% confluency, cells were pre-treated with various concentrations (10, 30, or 100 μg/mL) of TSE in serum-free media for 1 h, then treated with or without 100 ng/mL of LPS for further incubation for 30 min or 23 h. The LPS pre-treatment time schedule is mentioned in Supplementary Figure S1. An equal volume of vehicles were administered to the control and toxin groups. Quercetin, which is reported to exert potent anti-inflammatory activity, was used as the positive control [24].

### 4.5. Measurement of Cell Viability and Extracellular NO

Cell viability and NO levels were measured using a previously described method after 23 h of LPS stimulation [24]. The supernatant of the cell culture was harvested and mixed with an equal volume of Griess reagent (1% sulfanilamide, 0.1% naphthylethylenediamine dihydrochloride, and 2% phosphoric acid). After 10 min, the absorbance at 540 nm was measured using a microplate reader (Versamax, Molecular Devices, LLC, Sunnyvale, CA, USA). Sodium nitrite was used as a standard to calculate the NO^2−^ levels. Meanwhile, cell viability was measured by incubating cells with 1 mg/mL MTT for 4 h at 37 °C. After the MTT formazan was dissolved in dimethyl sulfoxide, the absorbance was measured on a microplate reader at 570 nm.

### 4.6. RNA Isolation and Quantitative Reverse-Transcription Polymerase Chain Reaction

mRNA transcription of cytokines was analyzed by quantitative reverse-transcription polymerase chain reaction (qRT-PCR). Using the Hybrid-R™ (GeneAll, Seoul, Korea), we extracted total RNA from the BV2 microglial cells, and measured the concentration using a NanoDrop ND-2000 spectrophotometer (Thermo Fisher Scientific Inc., Waltham, MA, USA). Next, 3 μg RNA samples were converted to cDNA using TOPscript™ RT DryMIX. The cDNA was analyzed by qRT-PCR using TOPreal™ qPCR 2× PreMIX (SYBR Green; Enzynomics) and the CFX Connect Real-Time PCR System (Bio-Rad Laboratories, CA, USA). Primers, synthesized at COSMO Genetech (Seoul, Korea), were as follows; *iNOS*: forward, 5′-GTG TTC TTT GCT TCC ATG CT-3′, reverse, 5′-AGT TGC TCC TCT TCC AAG GT-3′; *TNF-α*: forward, 5′-GAT TAT GGC TCA GGG TCC AA-3′, reverse, 5′-GCT CCA GTG AAT TCG GAA AG-3′; *IL-1β*: forward, 5′-CCC AAG CAA TAC CCA AAG AA-3′, reverse, 5′-GCT TGT GCT CTG CTT GTG AG-3′; *IL-6*: forward, 5′-CCG GAG AGG AGA CTT CAC AG-3′, reverse, 5′-TTG CCA TTG CAC AAC TCT TT-3′; *IL-10*: forward, 5′-AAG GCC ATG AAT GAA TTT GA-3′, reverse, 5′-TTC GGA GAG AGG TAC AAA CG-3′; *FIZZ1*: forward, 5′-TCC AGC TAA CTA TCC CTC CAC TGT-3′, reverse, 5′-GGC CCA TCT GTT CAT AGT-3′; *Ym-1*: forward, 5′-AGA GCA AGA AAC AAG CAT GG-3′, reverse, 5′-CTG TAC CAG CTG GGA AGA AA-3′; *HO-1*: forward, 5′-GCA CCG GCC GGA TGG AGC GTC C-3′, reverse, 5′-CGT CTC GGG TCA CCT GGC CCT TCT G-3′; *GAPDH*: forward, 5′-TGA ATA CGG CTA CAG CAA CA-3′, reverse, 5′- AGG CCC CTC CTG TTA TTA TG-3′.

### 4.7. Western Blot Analysis

BV2 cells were harvested 30 min after LPS stimulation to analyze NF-κB, and 23 h after LPS stimulation to analyze the other proteins [52]. Then, the cells were lysed in RIPA buffer containing protease/phosphatase inhibitors for whole protein analysis. All protein samples were stored at −80 °C before use. Cell lysates were separated by sodium dodecyl sulfate-polyacrylamide gel electrophoresis, and transferred to PVDF membranes. The membranes were then incubated overnight with 5% skim milk and primary antibodies. Next, the anti-rabbit HRP-conjugated secondary antibody was incubated for 1 h. Immunoreactive bands were detected using the ECL reagent. The visualization and quantitative assessments of band intensity were performed using the Image Lab Software (Bio-Rad Laboratories, Hercules, CA, USA).

### 4.8. Animals

Male ICR mice (7 weeks old) were purchased from Daehan Biolink (Eumseong, Korea), and housed at a constant temperature (23 ± 1 °C), humidity (60 ± 10%), and a 12 h light/dark cycle. In addition, the animals had free access to food and water. The mice were acclimated to their surroundings for 7 days, and kept under the same conditions before the start of the study.

### 4.9. Experimental Design of the Animal Study

Fifteen mice were randomly assigned to three groups: control (*n* = 5), LPS (*n* = 5), and LPS + TSE 50 mg/kg (*n* = 5). TSE dissolved in 2% Tween 80 was administered by oral gavage at 50 mg/kg for 5 days. Meanwhile, an equivalent volume of the vehicle was administered to the control and LPS groups. On the fifth day, 5 mg/kg LPS was intra-peritoneally injected 1 h after the administration of TSE. The mice were sacrificed 3 h after LPS injection.

### 4.10. Brain Tissue Preparation

For immunohistochemistry, the mice were transcardially perfused with 0.05 M phosphate buffered saline (PBS), and then fixed with 4% paraformaldehyde in 0.1 M phosphate buffer. Brains were removed, post-fixed overnight at 4 °C, and then immersed in 30% sucrose solution in PBS for cryoprotection. Serial 25 μm-thick coronal sections were cut on a freezing sliding microtome (Leica Microsystems Inc., Nussloch, Germany), and stored in a cryoprotectant (25% ethylene glycol, 25% glycerol, and 0.05 M phosphate buffer) at 4 °C until use.

### 4.11. Immunohistochemistry

The sections of the hippocampus and cerebral cortex subregions were selected according to the mouse brain atlas, from −1.94 to −2.30 mm following coordinates from the bregma. Free-floating brain sections were washed in PBS, and treated with 1% hydrogen peroxide for 15 min. The sections were then incubated with goat anti-Iba-1 overnight at 4 °C in the presence of 0.3% Triton X-100. After washing in PBS, the sections were incubated with biotinylated anti-goat IgG for 1 h, and with an ABC mixture for 1 h at room temperature. Peroxidase activity was visualized by incubating the sections with 3,3-diaminobenzidine in 0.05 M Tris buffer. After several washings with PBS, the sections were mounted on gelatin-coated slices, dehydrated, and cover-slipped using the histomount medium. Images were obtained using an optical bright-field microscope (Olympus Microscope System BX51; Olympus, Tokyo, Japan). Iba-1-positive cells in the cortex and hippocampus were quantified by stereological counting, and analyzed using the Image J software.

For Iba-1 + HO-1 double staining, the brain sections were incubated with a goat anti-Iba-1 overnight at 4 °C, and then with chicken anti-goat Alexa 488 for 1 h at room temperature. After rinsing with PBS, the sections were incubated with a rabbit anti-HO-1 overnight at 4 °C, and then with goat anti-rabbit Alexa Fluor 594 for 1 h at room temperature. The sections were mounted with an anti-fade fluorescent mounting medium containing DAPI. The images were captured using a K1-Fluo confocal microscope (Nanoscope Systems, Daejeon, Korea).

### 4.12. Statistical Analysis

All statistical parameters were calculated using GraphPad Prism 8.0 software (Graphpad Software, San Diego, CA, USA). Values were expressed as the mean ± standard error of the mean (S.E.M.), and analyzed using one-way analysis of variance (ANOVA), followed by Dunnett’s post hoc test. Differences with a *p* value less than 0.05 were considered statistically significant.

## Data Availability

The data that support the findings of this study are available from the corresponding author, upon reasonable request.

## Supplementary Materials

The following are available online at https://www.mdpi.com/article/10.3390/toxins13120898/s1, Figure S1: Effect of TSE on NO production and cell viability in BV2 microglia cells under LPS pre-treatment conditions, Figure S2: UPLC chromatograms of 3,29-dibenzoyl karounitriol and TSE.
