# Supplementary material for: Trichosanthis Semen Suppresses Lipopolysaccharide-Induced Neuroinflammation by Regulating the NF-κB Signaling Pathway and HO-1 Expression in Microglia
Source: Toxins (Basel). 2021 Dec 14;13(12):898. doi: 10.3390/toxins13120898 (PMC8704237; doi:10.3390/toxins13120898)
Supplement: Supplementary file 1 [file toxins-13-00898-s001.zip › toxins-1515007-supple for conversion update.pdf]

toxins

Supplementary Information

Trichosanthis Semen Suppresses Lipopolysaccharide-Induced Neuroinflammation by Regulating the NF-κB Signaling Pathway and HO-1 Expression in Microglia

Seungmin Lee [1,†], In Gyoung Ju [2,†], Yujin Choi [1], Sangsu Park [3], and Myung Sook Oh [1,2,4,*]

1 Department of Biomedical and Pharmaceutical Sciences, Graduate School, Kyung Hee University, Seoul 02447, Korea; smlee0817@khu.ac.kr (S.L.); yj001217@khu.ac.kr (Y.C.)
2 Department of Life and Nanopharmaceutical Sciences, Graduate School, Kyung Hee University, Seoul 02447, Korea; igju801@khu.ac.kr
3 Department of Fundamental Pharmaceutical Sciences, Graduate School, Kyung Hee University, Seoul 02447, Korea; x-zara@nate.com
4 Department of Oriental Pharmaceutical Science, College of Pharmacy and Kyung Hee East-West Pharmaceutical Research Institute, Kyung Hee University, Seoul 02447, Korea
* Correspondence: msohok@khu.ac.kr; Tel.: +82-2-961-9436; Fax: +82-2-963-9436
† These authors contributed equally to this work.

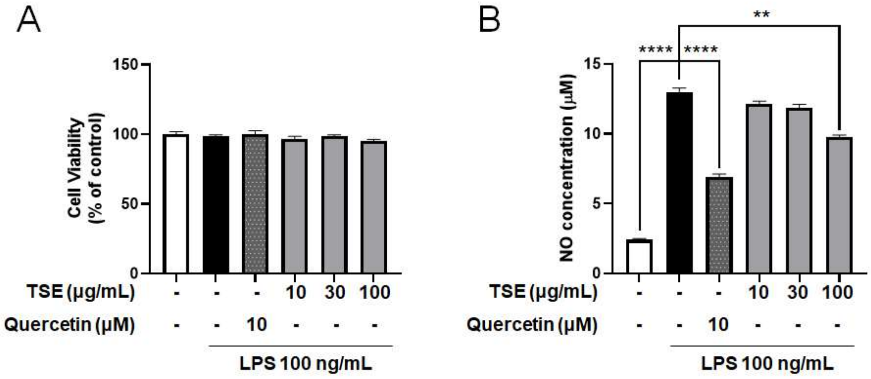

Figure S1. Effect of TSE on NO production and cell viability in BV2 microglia cells under LPS pretreatment conditions. The cells were treated with different concentrations of TSE (10, 30, or 100 μg/mL) or quercetin (10 μM) for 9 h after LPS treatment for 6 h. Cell viability was measured by MTT assay (**A**). The cell culture supernatant containing NO was evaluated by Griess reagent colorimetric reaction ($n = 3$ per group) (**B**). Data were analyzed by one-way ANOVA, followed by Dunnett's post hoc test. **$p < 0.01$ and ****$p < 0.0001$.

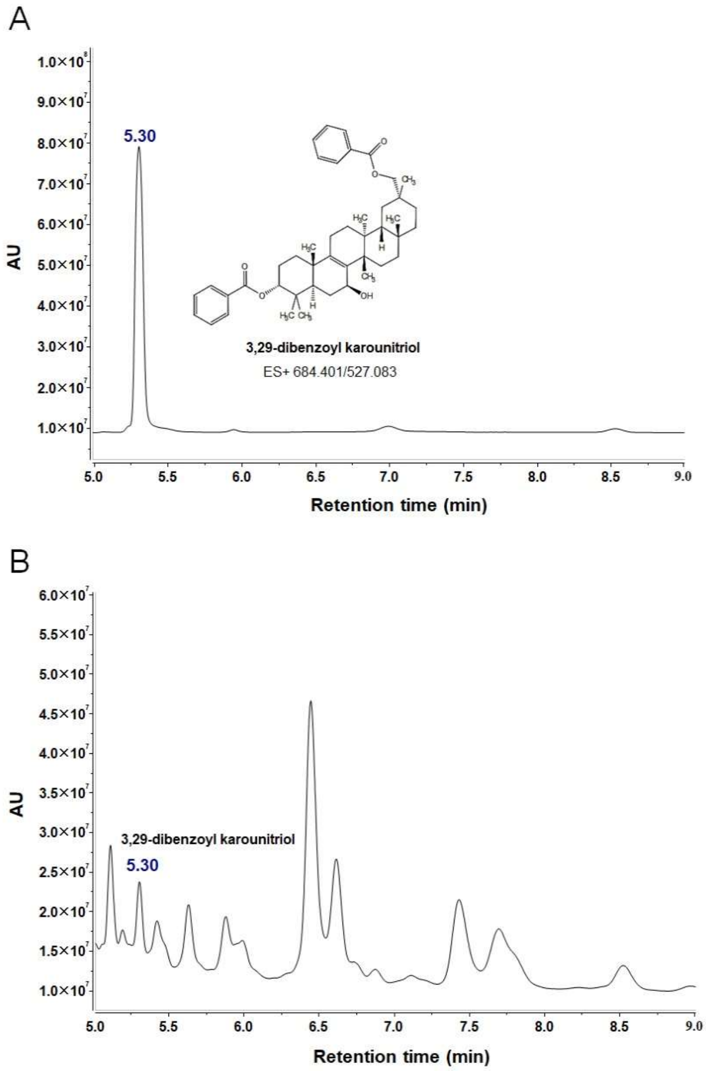

Figure S2. UPLC chromatograms of 3,29-dibenzoyl karounitriol and TSE. The chromatograms of 3,29-dibenzoyl karounitriol (**A**) and TSE (**B**) were detected at total absorbance chromatogram.
